# Determinants of risky sexual behaviour among undergraduate students at the University of Gondar, Northwest Ethiopia

**DOI:** 10.1017/S0950268821002661

**Published:** 2021-12-09

**Authors:** Esayas Alemshet Tekletsadik, Aynalem Adu Ayisa, Enyew Getaneh Mekonen, Belayneh Shetie Workneh, Mohammed Seid Ali

**Affiliations:** 1 Department of Surgical Nursing, School of Nursing, College of Medicine and Health Sciences, University of Gondar, Gondar, Ethiopia; 2 Department of Emergency and critical care Nursing, School of Nursing, College of Medicine and Health Sciences, University of Gondar, Gondar, Ethiopia; 3 Department of Pediatrics and Child Health Nursing, School of Nursing, College of Medicine and Health Sciences, University of Gondar, Gondar, Ethiopia

**Keywords:** Determinants, Northwest Ethiopia, prevalence, risky sexual behaviour, undergraduate students

## Abstract

Risky sexual behaviour (RSB) is defined as behaviours leading to sexually transmitted diseases and unintended pregnancies. According to the Joint United Nations Program on HIV/AIDS, HIV infection was very high among adolescents and youths living in sub-Saharan Africa including Ethiopia. This study was aimed to assess the prevalence of RSB and associated factors among undergraduate students at the University of Gondar.

An institution-based cross-sectional study was conducted from June to July 2019 and a simple random sampling technique was employed to select 420 students. Data were collected using a structured self-administered questionnaire, entered into Epi-info version 7.0 and exported to Statistical Package for Social Sciences (SPSS) version 25 for analysis, and presented in frequencies, percentages and tables. Bivariable and multivariable logistic regression analysis were carried out to identify variables having significant association with RSB.

The prevalence of RSB among undergraduate students at the University of Gondar was 44.0%. Age [adjusted odds ratio (AOR): 2.12; 95% confidence interval (CI) (1.19–3.79)], residence [AOR: 2.14; 95% CI (1.22–3.75)], living arrangement [AOR: 9.79; 95% CI (5.34–17.9)], daily religious attendance[AOR: 0.57; 95% CI (0.33–0.99)], drink alcohol [AOR: 9.19; 95% CI (3.74–22.59)] and having information about reproductive health and sexually transmitted diseases [AOR:3.05; 95% CI (1.00–9.27)] were factors significantly associated with RSB.

Nearly half of the respondents engaged in risky sexual activity. This prevalence is high and the students are at high risk of exposure to sexually transmitted diseases that need reproductive health intervention like counselling and discussion. Creating awareness is needed for the students regarding reproductive health and the risk of sexually transmitted diseases. In addition, giving special attention is required for students who use alcohol, who did not live with family and who have urban residence.

## Background

Risky sexual behaviour (RSB) is defined as any sexual activity that increases the risk of acquiring sexually transmitted infections (STI) and unwanted pregnancies [1]. RSB is a major public health problem around the globe [2]. RSB includes having sex with multiple sexual partners, not using or inconsistent condom use, sexual intercourse under the influence of substance use [3]. If not appropriate interventions exist, unsafe behaviours can expose them to a higher risk of acquiring HIV, other STIs and unwanted pregnancy [4].

Globally, young people (15–35 years of age) are the most susceptible groups for RSB and account for an estimated 45% of new HIV infections [5]. RSB can be aggravated by low income, job insecurity, lack of awareness about sexual and reproductive health issues and harmful traditional practices [6]. University students are viewed as being at higher risks to acquire HIV infection or STI and they are categorized under the most at-risk population due to their engagement in RSB and their sense of non-vulnerability [7].

It is considered that University and College students are aware of HIV risks and preventive mechanisms; however, evidence showed that they are usually engaged in higher in RSB [8]. Promoting safe sexual activity would contribute to the reduction of sex-related morbidity and mortality caused by unsafe abortion and HIV. Unprotected sexual practice and the associated exposure to infection is one of the major causes of preventable mortality in low and middle-income countries. It is the major mode of transmission for HIV and human papillomavirus, about the mortality of more than one million people worldwide [9].

According to the result of different studies, the extent of RSB among university students in Africa is high. The prevalence of RSB was 26% in Uganda [10], 63.9% in Botswana [11] and 63% in Nigeria [12].

In Ethiopia, youth people represented one of the country's largest groups, comprising about 35% of the population, and University students are exposed to RSB such as unsafe sex leading to HIV infection and other STIs as well as unwanted pregnancies [13]. Identifying the determinant factors of RSB among University students is also an important indicator for the rest young population to take appropriate preventive measures [14]. They are more susceptible to wider sexual and reproductive health problems due to the new environment in the universities with poor protection [15]. Age and the need to explore life, financial benefit, substance use, peer pressure and absence of preventive strategies and living independently away from home were considered important factors for RSB [13].

There are studies about University students' in African countries indicating a higher prevalence of RSB [16]. However, the determinant factors of RSB among University students are not well known in the study area. RSB such as having multiple sexual partners and sexual intercourse without a condom with a non-regular partner is a common practice [17]. Therefore the purpose of this study was to assess the prevalence and determinant factors of RSB among University of Gondar undergraduate students, Northwest Ethiopia.

## Method and materials

### Study design and period

An institution-based cross-sectional study was conducted from June to 30 July 2019.

### Study area

The study area was at the University of Gondar which is located 750 km far from Addis Ababa, the capital city of Ethiopia. It is one of the first generation universities in Ethiopia and was established as a public health college in 1954.

### Study participants

Those students who were available during the data collection period were included in the study. While those students who were not present during the data collection period were excluded from the study.

### Sample size determination and sampling technique

The sample size was calculated using the single population proportion formula by taking the estimated proportion of RSB 46.6% from a study conducted in southwest Ethiopia [3], 95% confidence interval (CI) and a 5% margin of error. The final sample size was 420 after adding a 10% non-response rate. The proportional allocation formula was used to allocate the number of students to the five campuses. Then the sampling frame was prepared for each campus by having lists of students from the main registrar's office. Finally, the study participants were selected using a simple random sampling technique.

### Operational definitions

#### Risky sexual behaviours

Having more than one sexual partner or inconsistent condom use/not using a condom during sex in a lifetime.

### Data collection tools and procedure

A structured pre-tested self-administered questionnaire was used to collect the data. The tool was arranged into four categories; the first part of the questionnaire contains the socio-demographic characteristics of the participants, the second part describes sexual experience-related variables, the third section states the sexual behaviour in the past 12 months, and the last section contains RSB related questions. There were three trained BSc nurse data collectors and two MSc nurse supervisors during data collection.

### Data quality control

Before the actual data collection period, the tool was pretested with 5% of the total sample size. Amendments on the instrument, such as unclear questions and ambiguous words made accordingly. The pretest was also be used to estimate how much time it takes to administer the entire questionnaire. The tool was first developed in the English language and translated to the Amharic language with back translation to English for consistency. The one-day training was given to data collectors and supervisors on the objective of the study, instrument and data collection procedures by the principal investigators. Supervision was conducted by the principal investigators and supervisors. To ensure data quality, each data collector checked the questionnaire from each study participant for completeness. The supervisors and principal investigators review each questionnaire daily and check for completeness.

### Data processing and analysis

Before the analysis data clean-up and cross-checking were done. Then the data were entered into Epi-info version 7.0 and exported to Statistical Package for Social Sciences (SPSS) version 25 for analysis [18]. Descriptive statistics like frequencies, percentages and tables were used to present data. To assess the association between the different independent variables (Age, marital status, residence, mother's education, living arrangement, daily religious attendance, chew chat, drink alcohol, ever see pornography and having information about reproductive health and sexually transmitted diseases) with the dependent variable (RSB), first, bivariable relationships between each independent variable and outcome variable were investigated using a binary logistic regression model. Those independent variables with a *P*-value <0.2 at the bivariable level were eligible for multivariable analysis to control potential confounding factors. After adjusting their effect on the outcome variable, those variables with a *P*-value < 0.05 with a 95% CI were considered as significantly associated with RSB.

### Ethical consideration

Ethical clearance was sought from the University of Gondar, School of Nursing. From the campus dean's office, a written letter was taken. The participants were informed and written consent was obtained from each study participant. Omitting direct personal identifiers on the questionnaire, by using code numbers, storing data locked with a password and not misusing or wrongfully disclosing their information were considered to maintain confidentiality. Study participants were also informed that involvement was a volunteer and they can take out from the study involvement at any step if they are not happy with the study.

## Results

### Socio-demographic characteristics of respondents

A total of 382 undergraduate students participated in this study, with a response rate of 91%. More than half (53.1%) of the respondents were male. Nearly three fourth (71.5%) of the respondents were under the age category of less than 24 years with the age range of between 18 and 34 years. Concerning their residence more than half (63.6%) of the respondents live in an urban area. Regarding their living arrangement, more than half (55.8%) of the respondents did not live with their families (Table 1).

**Table 1.** Socio-demographic characteristics of undergraduate students at the University of Gondar, Northwest Ethiopia, 2019 (*n* = 382)

Variables	Category	Frequency	Percent
Age	<24	273	71.5
	>24	109	28.5
Sex	Male	203	53.1
	Female	179	46.9
Marital Status	Single	325	85.1
	Married	57	14.9
Residence	Urban	243	63.6
	Rural	139	36.4
Previous high school	Private school	66	17.3
	Missionary/religious school	4	1
	Others	312	81.7
Father's education	Unable to read and write	107	28
	Read and write	201	52.6
	College/University degree and above	74	19.4
Mother's education	Unable to read and write	181	47.4
	Read and write	146	38.2
	College/University degree and above	55	14.4
Living arrengment	Live without family	213	55.8
	Live with family	169	44.2
Daily religious attendance	Yes	255	66.8
	No	127	33.2
Year of education	First-year	57	14.9
	Second-year	70	18.3
	Third-year	123	32.2
	Fourth-year	132	34.6

### Sexual experience of the respondents

Of the total study participants majority, 196 (51.8%) reported having a history of previous sexual intercourse, among those 34.2% of them made their first sexual intercourse before 18 years old. The majority 126 (64.29%) of the study participants made their first sexual intercourse before joining the university, and 94 (24.6%) of them made their first sexual intercourse with their boy/girlfriends. About 68 (17.8%) of them had sex with a commercial sex worker (Table 2).

**Table 2.** Sexual experience of undergraduate students at the University of Gondar, Northwest Ethiopia, 2019 (*n* = 382)

Variables	Category	Frequency	Percent
Have you ever had sexual intercourse	Yes	196	51.8
	No	184	48.2
Age at first sex	⩽18 years	67	34.18
	>18 years	129	65.8
When do you make your first sexual intercourse	Before joining university	126	64.29
	After joining university	70	35.71
With whom you made your first sexual intercourse?	Boy girlfriend	94	24.6
	Causal boy/girlfriend	36	9.4
	Husband/wife	58	15.2
	Commercial sex worker	8	2.1
What is your reason for making your first sexual intercourse	Fall in love	91	23.8
	Had personal desire	42	11
	Marital	48	12.6
	Raped	3	0.8
	Peer pressure	11	2.9
	Influence of alcohol	1	0.3
Age of the person you did intercourse with	Similar age	104	27.2
	Younger than me	52	13.6
	More than 10 years old	8	2.1
	Less than 5 years	19	5
	5–10 years old	13	3.4
How many times have you had sex with commercial sex workers	One only	47	12.3
	Two only	12	3.1
	Three or more	9	2.4

### Substance use and health risk behaviours of the students

Among the total respondents, seventy-nine (20.7%) of them were alcohol users and seventy-nine (20.7%) of the students were chat chewers. Seventy-four (19.4%) of the students were cigarette smokers. One-fifth (20.6%) of the respondents had seen pornographic movies in their life. Eighty-seven (22.8%) of the students were going to the nightclub. One hundred thirty-one (34.3%) of the students had a partner who influence sexual intercourse (Table 3).

**Table 3.** Substance use and health risk behaviours of undergraduate students at University of Gondar, Northwest Ethiopia, 2019 (*n* = 382)

Variables	Category	Frequency	Percent
Do you Chew chat?	Yes	79	20.7
	No	303	79.3
When do you start chewing chat?	Before joining the University	33	8.6
	After joining the University	46	11.6
Have you ever used Alcohol?	Yes	79	20.7
	No	303	79.3
Have you ever smoked a cigarette?	Yes	74	19.4
	No	308	80.6
When do you start smoking a cigarette?	Before joining the University	23	6
	After joining the University	51	13.4
Did your partners influence you to use substances?	Yes	113	29.6
	No	269	70.4
Ever seen pornographic movies?	Yes	77	20.2
	No	305	79.8
Ever went to a nightclub?	Yes	87	22.8
	No	295	77.2
Do you have partner influence for sexual intercourse	Yes	131	34.3
	No	251	65.7

### Prevalence of risky sexual behaviour

Among the total participants who had a history of sexual intercourse, more than half (53.6%) of the respondents were never used a condom during sex. Regarding their sexual partner, nearly half (48.5%) of the respondents had more than one sexual partner. The overall prevalence of RSB of undergraduate students of the University of Gondar was 44.0% with 95% CI (38.7–49.2%) (Table 4).

**Table 4.** Prevalence of RSB of undergraduate students at the University of Gondar, Northwest Ethiopia, 2019 (*n* = 196)

Variables	Category	Frequency	Percent
Do you use a condom during sex	Always	37	18.9
	Some times	54	27.5
	Never	105	53.6
Do you have more than one sexual partner	Yes	82	48.5
	No	114	51.5

### Factors associated with risky sexual behaviour

In bivariable logistic regression analysis, age, marital status, residence, mother's education, living arrangement, daily religious attendance, chew chat, drink alcohol, ever see pornography and having information were found to be significantly associated with RSB. Using multivariable logistic regression analysis, age, residence, living arrangement, daily religious attendance, drinking alcohol and having information were significantly associated with RSB. Those students in age group greater than 24 years were 2.12 times more likely to be engaged in RSB as compared to the younger age group of the students [adjusted odds ratio (AOR): 2.12; 95% CI (1.19–3.79)]. Those students who lived in urban areas were almost 2.14 times more likely to be engaged in RSB as compared to those students who lived in rural areas [AOR: 2.14; 95% CI (1.22–3.75)]. Students who lived without family or were not controlled by family members were more than 9.79 times more likely to be engaged in RSBs as compared to those students who lived with family members or controlled by family members [AOR: 9.79; 95% CI (5.34–17.9)]. Those study participants who reported to have alcohol are about nine times more likely to have RSB as compared with those study participants who reported not to have alcohol[AOR: 9.19; 95% CI (3.74–22.59)].

The odds of having RSB is about 43% less likely In study participants who have a daily religious attendance than study participants who have no daily religious attendance [AOR: 0.57; 95% CI (0.33–0.99)]. Moreover, those study participants who have information regarding reproductive health and sexually transmitted disease are about thre times more likely to have RSB as compared with respondents who have no information regarding RSB [AOR:3.05; 95% CI (1.00–9.27)] (Table 5).

**Table 5.** Bivariable and multivariable logistic regression analysis of factors associated with RSB among undergraduate students at the University of Gondar, Northwest Ethiopia, 2019 (*n* = 382)

Variables	Category	Risky sexual behaviour		COR 95% CI	AOR 95% CI	*P*-value
		Yes	No			
Age	<24	110	163	1	1	1
	>24	58	51	**1.69 (1.08–2.64)**	**2.12 (1.19–3.79)**	**0.011**
Marital status	Single	124	201	**1.77 (1.09–2.88)**	0.61 (0.31–1.19)	0.145
	Married	44	13	1	1	1
Residence	Urban	119	124	**1.76 (1.15–2.71)**	**2.14 (1.22–3.75)**	**0.008**
	Rural	49	90	1	1	1
Mothers education	Illiterate	81	100	1.54 (0.82–2.88)	1.89 (0.82–4.38)	0.133
	Read and write	68	78	1.65 (0.87–3.15)	1.85 (0.81–4.24)	0.143
	College and above	19	36	1	1	1
Living arrangement	With family	45	140	1	1	1
	Without family	123	74	**5.17 (3.32–8.05)**	**9.79 (5.34–17.9)**	**<0.001**
Daily religious attendance	Yes	100	155	**0.56 (0.36–0.86)**	**0.57 (0.33–0.99)**	**0.049**
	No	68	59	**1**	1	1
Chew chat	Yes	90	50	**3.79 (2.44–5.87)**	1.39 (0.71–2.71)	0.331
	No	78	164	1	1	1
Drink alcohol	Yes	66	13	**10.01 (5.27–18.98)**	**9.19 (3.74–22.59)**	**<0.001**
	No	102	201	1	1	1
Ever see pornography	Yes	54	23	3.93 (2.29–6.75)	2.04 (0.95–4.38)	0.067
	No	114	191	1	1	1
Having information	Yes	161	197	1.98 (0.80–4.90)	**3.05 (1.00–9.27)**	**0.049**
	No	7	17	1	1	1

*Note:* Significant variables are provided in bold.

## Discussion

The result of the current study revealed that the prevalence of RSB is 44.0%. It implies that RSB is a common health concern among undergraduate university students which needs special consideration from youth-friendly service givers, policymakers, university administrators and families. The finding of this study is in line with studies conducted in Jimma Ethiopia 43.5% [19] Addis Ababa Ethiopia 43.1% [20], Wolyita Ethiopia 46.0% [21], Lalibela Ethiopia 46.5% [22]. The prevalence of RSB in this study is higher than studies conducted in Arba Minch 22.4% [23], Siri Lanka 12.4% [24]. The possible reason for this difference might be due to the difference in year of study, sample size and definition of RSB used in each study. However, it is lower than studies conducted in Portugal 64.0% [25], China 49.4% [26]. The possible justification for this difference might be that due to the variation in socio-cultural difference. On the other hand, it might be due to the fear of becoming infected with sexually transmitted diseases like HIV. Meaning in developing countries the risk of exposing to STD is high compared with developed countries.

Among the determinant factors, the age of the participants was one of the factors significantly associated with RSB. Those students with ages greater than 24 years were about two times more likely to be engaged in RSB as compared to the younger age group of the students. This finding was supported by the study conducted in Addis Ababa, Ethiopia [20]. This might be due to the age group of students greater than 24 years who were commonly called sexually active and in the fire age group because their sexual desire increased as they became physically and physiologically matured.

This study also showed that place of residence was another determinant factor associated with sexual behaviour. Those students who lived in urban areas were almost two times more likely to be engaged in RSB as compared to those students who lived in rural areas. This finding was supported by other studies, which revealed that those study participants who had urban residency are more likely to engage in RSB [27–30]. This might be due to that students from the urban area had access to mobile communication, online chatting, seeing pornography and sex film in the cinema or on the internet which easily initiated their sexual desire and engaged in RSB, but students from the rural area might be less exposed to sex initiating situations. This might also be due to there is a culture in rural Ethiopia, sex before marriage is morally not acceptable.

Another factor associated with RSB was living arrangements. Students who did not live with family were more than nine times more likely to be engaged in RSBs as compared to those students who lived with the family. This finding was in agreement with other studies conducted in different countries [29, 31, 32]. This might be due to students, who lived without family feeling free to have sexual contact with an opposite-sex partner, but those students who lived with family were under the control of their family and they did not get free time for sexual contact.

The result of this study revealed that alcohol consumption and RSB are significantly associated. The odd of engaging in RSB is nine times higher in respondents who consume alcohol than respondents who didn't consume alcohol. This finding is supported by studies conducted in different countries [4, 19, 24, 33, 34]. The possible justification could be because having alcohol will cause intoxication which leads university students to have more sexual arousal to commit RSB.

The finding of this study also revealed daily religious attendance and engaging in RSB are inversely associated. Those students who reported to have daily religious attendance are about 43% less likely to engage in RSB as compared with university students who reported not to have daily religious attendance. This finding is in agreement with other studies [35, 36]. The possible justification could be because those study participants who reported to have daily religious attendance might be more prone to follow religious commandments so that they might not have alcohol and other substances. Therefore, they might be less likely to engage in RSB.

Moreover, the study revealed that having information regarding reproductive health and sexually transmitted diseases is significantly associated with RSB. Study participants who have information regarding reproductive health and sexually transmitted disease are about three times more likely to have RSB as compared with respondents who have no information regarding reproductive health and sexually transmitted disease. Logically, it is expected to be less RSB with participants having information about reproductive health and RSB. But in our finding, participants having information were more likely to have RSB. It might be due to ignorance and limitation of the study design. Whether the act of RSB or information regarding RSB comes first is not identified. Furthermore, participants with RSB may seek more information after they engaged in RSB due to fear of its negative outcomes.

## Conclusion

Nearly half of the respondents engaged in risky sexual activity. This prevalence is high and the students are at high risk for exposure to sexually transmitted diseases that need reproductive health intervention like counselling and discussion. Creating awareness for the students regarding reproductive health and the risk of sexually transmitted diseases is needed. In addition, giving special attention is required for students who use alcohol, who did not live with family, and who have urban residence.

## Data Availability

All data are available upon reasonable request.
